# P-1276. Infant Treatment for Congenital Syphilis Among Syphilis-Impacted Pregnancies

**DOI:** 10.1093/ofid/ofae631.1457

**Published:** 2025-01-29

**Authors:** Christina L Sancken, Jeffrey Carlson, Sara L Schubert, Kevin P O’Callaghan, Alison Fountain, Kate Russell Woodworth

**Affiliations:** CDC, Atlanta, Georgia; CDC, Atlanta, Georgia; CDC, Atlanta, Georgia; Centers for Disease Control and Prevention, Powder Springs, GA; CDC, Atlanta, Georgia; CDC, Atlanta, Georgia

## Abstract

**Background:**

Congenital syphilis (CS) has been rising in the U.S. since 2013 and is a significant public health challenge^1^. The Sexually Transmitted Infections (STI) Treatment Guidelines provide treatment recommendations for four different clinical scenarios based on adequacy of treatment during pregnancy, laboratory testing of both the infant and birth parent, and clinical signs^2^. We used data from the Surveillance for Emerging Threats to Pregnant People and Infants Network^3^ from five U.S. state and local health departments to describe patterns of treatment among infants born to people with syphilis during pregnancy (Figure 1).

**Figure:**
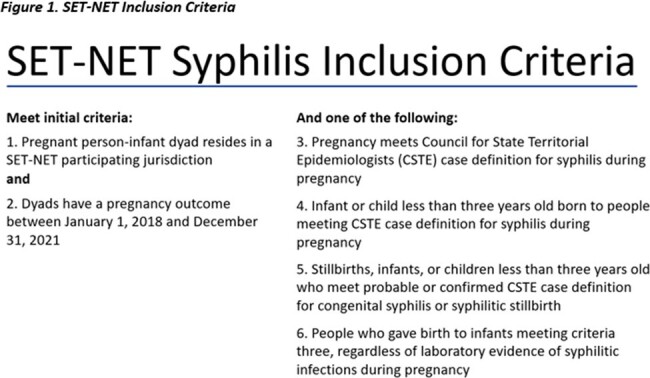

**Methods:**

Infants were classified based on modified STI Treatment Guidelines clinical scenarios^2^, and included three of the four clinical scenarios: confirmed/highly probable CS, possible CS, and CS less likely. We describe the frequency of prenatal and neonatal characteristics among those who did and did not receive treatment as recommended by the STI Treatment Guidelines (Figure 2).

**Figure:**
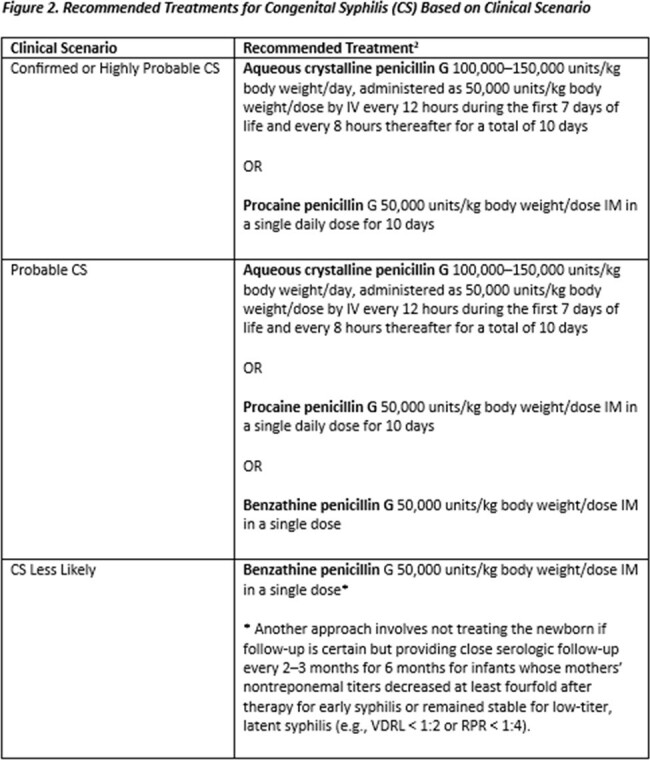

**Results:**

Of the 1368 liveborn infants with complete medical record abstraction, 13% were classified as confirmed/highly probable CS, 33% as possible CS, and 54% as CS less likely (Figure 3). Frequency of receiving the standard of care was 65% for confirmed/highly probable, 76% for possible, and 34% for CS less likely. The frequency of no reported treatment was 16% for confirmed/highly probable CS, 20% for possible CS, and 45% for CS less likely. The frequency of no reported treatment among confirmed/highly probable CS was highest among infants born to people who were Black non-Hispanic, though numbers were small. Of infants with CS less likely, 18% received more than the recommended one dose (Table).

**Figure:**
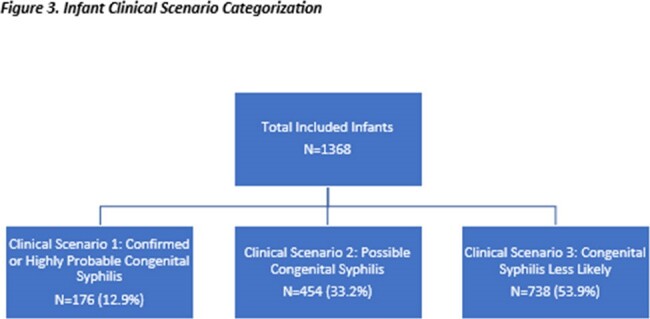

**Conclusion:**

This report highlights opportunities for improvement in care of infants exposed to syphilis, including racial disparities in recommended care^4^, that require multipronged approaches to address. Provider education and strong partnerships with well-resourced public health systems can improve coordination of care to ensure infants receive the standard of care. Efforts to improve care of syphilis exposed infants must include efforts to better address larger issues of systematic racism and historical injustices in healthcare and public health to improve equity.

**Table.**
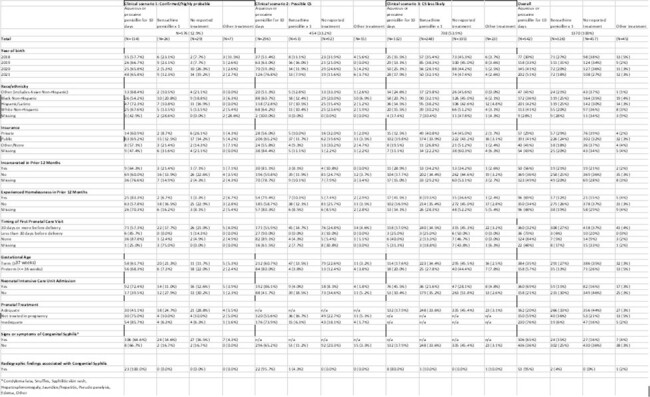
Cohort Characteristics by Clinical Scenario and Infant Treatment Status

**Disclosures:**

**All Authors**: No reported disclosures

